# Long-Term Exposure of Mice to Nucleoside Analogues Disrupts Mitochondrial DNA Maintenance in Cortical Neurons

**DOI:** 10.1371/journal.pone.0085637

**Published:** 2014-01-20

**Authors:** Yulin Zhang, Fengli Song, Ziyun Gao, Wei Ding, Luxin Qiao, Sufang Yang, Xi Chen, Ronghua Jin, Dexi Chen

**Affiliations:** 1 Department of Infectious Diseases, Beijing You An Hospital, Capital Medical University, Beijing, China; 2 Department of Neurosurgery, The Second Affiliated Hospital of Nanchang University, Nanchang, Jiangxi Province, China; 3 Department of Otolaryngology, The First Affiliated Hospital Of Nanjing Medical University, Nanjing, China; Medical University of South Carolina, United States of America

## Abstract

Nucleoside analogue reverse transcriptase inhibitor (NRTI), an integral component of highly active antiretroviral therapy (HAART), was widely used to inhibit HIV replication. Long-term exposure to NRTIs can result in mitochondrial toxicity which manifests as lipoatrophy, lactic acidosis, cardiomyopathy and myopathy, as well as polyneuropathy. But the cerebral neurotoxicity of NRTIs is still not well known partly due to the restriction of blood-brain barrier (BBB) and the complex microenvironment of the central nervous system (CNS). In this study, the Balb/c mice were administered 50 mg/kg stavudine (D4T), 100 mg/kg zidovudine (AZT), 50 mg/kg lamivudine (3TC) or 50 mg/kg didanosine (DDI) per day by intraperitoneal injection, five days per week for one or four months, and primary cortical neurons were cultured and exposed to 25 µM D4T, 50 µM AZT, 25 µM 3TC or 25 µM DDI for seven days. Then, single neuron was captured from mouse cerebral cortical tissues by laser capture microdissection. Mitochondrial DNA (mtDNA) levels of the primary cultured cortical neurons, and captured neurons or glial cells, and the tissues of brains and livers and muscles were analyzed by relative quantitative real-time PCR. The data showed that mtDNA did not lose in both NRTIs exposed cultured neurons and one month NRTIs treated mouse brains. In four months NRTIs treated mice, brain mtDNA levels remained unchanged even if the mtDNA levels of liver (except for 3TC) and muscle significantly decreased. However, mtDNA deletion was significantly higher in the captured neurons from mtDNA unchanged brains. These results suggest that long-term exposure to NRTIs can result in mtDNA deletion in mouse cortical neurons.

## Introduction

With the advent of highly active antiretroviral therapy (HAART), the incidence of HIV associated dementia (HAD) has drastically fallen, while the cumulative prevalence of HIV associated neurocognitive disorders (HAND) has risen [1]. Despite the decline of HAD incidence, neurological complications still remain an important cause of disability and death associated with AIDS [2]. The rise in the prevalence of HAND is consistent with the poor penetration of some of these agents into the CNS, hence increasing the longevity of the virus in the CNS [3], [4]. Cognitive impairment remained stable in one group of HAND patients, but progressive in other group even with effective antiretroviral treatment [5], [6]. It is still not well known whether this disparity is caused by increased survival of the virus in the brain or by antiretroviral agent neurotoxicity.

Nucleoside analogue reverse transcriptase inhibitor (NRTI), an integral component of HAART, is widely used to inhibit human immunodeficiency virus (HIV) replication. Since NRTIs cannot form 3′-phosphodiester bond themselves, they are phosphorylated by host cell enzymes to active triphosphates, which inhibit HIV reverse transcriptase and/or act as chain terminators when incorporated into viral DNA chain. HAART cannot eradicate HIV due to virus reservoir, drug resistance and other reasons, which leads to a lifelong antiviral therapy in HIV infected patients. However, long-term exposure to NRTIs can result in side effects similar to manifestations of a series of genetic mitochondria diseases, including lactic acidosis, hepatic steatosis, (cardio-) myopathy, pancreatitis, lipodystrophy, and probably polyneuropathy [7]. The mechanisms of these toxicities have been found to involve inhibition of DNA polymerase gamma leading to inhibition of mitochondria DNA (mtDNA) synthesis, resulting in oxidative stress and eventual mitochondrial dysfunction [8]–[10]. But the cerebral neurotoxicity of NRTIs which is restricted by blood-brain barrier (BBB) and microenvironment of the central nervous system (CNS) is still not clear.

In this study, in order to explore the possible role of NRTIs in HAND development, we intend to identify whether NRTIs can induce neuronal mitochondrial DNA loss in mice.

## Materials and Methods

### Animals and Materials

Experimental protocols were approved by the Institutional Animal Care and Use Committee and conformed to the Capital Medical University guidelines for the care and use of animals in research. Balb/C mice were obtained from Chinese Military Academy of Medical Sciences. Stavudine (D4T), zidovudine (AZT), lamivudine (3TC) and didanosine (DDI) were gifted from Northeast Pharmaceutical Group Co., Ltd (Shenyang, China). Propidium iodide (PI) was obtained from Sigma (St. Louis, MO). Calcein AM was obtained from Molecular Probes (Eugene, OR). Neurobasal medium, B27, penicillin-streptomycin and Glutamax used in neuronal cultures were obtained from Gibco (USA). Genome DNA extraction kit was obtained from QIAGEN China Co., Ltd. (Shanghai, China).

### Mice Exposure to Nucleoside Analogues

The powders of D4T, AZT, 3TC and DDI were dissolved in dimethyl sulfoxide (DMSO) and stored at −20°C. Then, high concentrations of D4T, AZT 3TC or DDI were rapidly diluted with double distilled water and injected to BALB/C mice (7 weeks of age, 28–30 g of weight) by intraperitoneal injection with D4T 50 mg/kg, AZT 100 mg/kg, 3TC 50 mg/kg or DDI 50 mg/kg per day, five days per week for one or four months. Five BALB/C mice were treated with each of the nucleoside analogues. Simultaneously, five BALB/C mice were injected with double distilled water as negative controls. The mice were maintained throughout the experiment on a 12/12-h light/dark cycle in a temperature- and humidity-controlled environment with food and water available *ad-libitum*.

### Laser Capture Microdissection (LCM)

Mice were sacrificed by cervical dislocation and cerebral cortex was rapidly dissected and frozen in liquid nitrogen. Fresh-frozen samples were embedded in optimum cutting temperature compound (OCT), 6-µm-thick sections were cut using a cryostat at −13°C and mounted on slides covered by polyethylene membrane (PEN slides; C. Zeiss, Thornwood, NY). Each slide was rinsed with 70% ethanol for 1 minute, and stained in Mayer’s hematoxylin (Sigma Chemical Co., St. Louis, MO) for 30 seconds. After twice rinsed with deionized water, the slide was treated with 70% ethanol for 1 minute, 95% ethanol for 1 minute, 1% Eosin Y in alcohol (Harleco) for 20 seconds, twice more with fresh 95% ethanol for 1 minute, twice with fresh 100% ethanol for 1 minute, then twice with fresh 100% Xylene for 1 minute. After air-drying for 5 minutes with the airflow turned on, the stained slides were microdissected within 2 hours. LCM was performed through a P.A.L.M. Robot-Microbeam System (Oberkochen, Germany) following the manufacturer’s recommendations and previous report [11]. First,the CapSure Macro LCM Caps and standard hematoxylin and eosin-stained coverslipped slides were put on the fixing devices respectively. Then they were moved to the work areas by joystick, and single neuron was found and signed at ×400 magnification. Last, the neuron was captured to the cap by clicking the button using the mouse.

### Primary Neuronal Cell Cultures and Nucleoside Analogues Toxicity Experiment

Primary cortical neuronal cultures were established from 16 days old embryonic BALB/C mice. Dissociated cells were plated onto poly-D-lysine coated plates (24 well plates, 2.5×10^5 ^cells/well or 12 well plates 6.0×10^5 ^cells/well or six well plates, 12×10^5 ^cells/well) in Neurobasal medium supplemented with 2% B27, 50U/50 *µ*g/mL penicillin-streptomycin and 2.0 mM Glutamax and grew at 37°C in 100% humidity, 95% room air/5% CO_2_. Cover glasses pretreated with acetone, absolute ethanol and 0.1 M HCl in turn were coated and used for immunofluorescence assay. Every three days, half of the mediums were replaced by fresh ones.

For nucleoside analogues toxicity experiment, 25 µM D4T, 50 µM AZT, 25 µM 3TC, or 25 µM DDI were tested to be the appropriated mtDNA toxicity concentrations which did not inhibit neuron growth by PI and Calcein-AM uptake test and were kept in the cultural medium for seven days respectively.

### Assessment of Neuron Growth

Cell viability was assessed by PI and Calcein-AM uptake test using the Nikon inverted microscope (Nikon Eclipse TE200). Cultured neurons were incubated with 1 *µ*g/mL Calcein/AM for 30 min; 10 *µ*g/mL PI was then added for 15 min in medium at 37°C. For cell counting, cells were visualized using a Nikon 40× objective lens. In a blinded fashion, three images per well (24 wells) were generated and three wells for each condition were analyzed. Cell mortality was expressed as the ratio of PI-positive cells/Calcein positive+PI positive cells.

### DNA Isolation and Quantitative Real-time PCR

DNA from tissues and LCM cells were isolated using a kit from QIAGEN China Co., Ltd. (QIAGEN, Shanghai, China) following the recommended protocol. Quantitative PCR (qPCR) assays were performed as previously described with minor modifications [12]. The TaqMan 7900 HT system was used to perform real-time PCR amplification of the mtDNA regions using the following primers and probes: Forward Primer for Cox II: 5′-cgacctaaaacctggtgaacta-3′, Reverse Primer for Cox II: 5′-ttggaagttctattggcagaac-3′, Probe for Cox II: 5′-FAM-actgctagaagttgataaccgagtc-TAMRA-3′; Forward Primer for ND1: 5′-atatcctaacactcctcgtcc-3′, Reverse Primer for ND1: 5′-agggccttttcgtagttg-3′, Probe for ND1: 5′-FAM-ttctaatcgccatagccttcctaac-TAMRA-3′; Forward Primer for ND4: 5′-aatatattctcctcagacccc-3′, Reverse Primer for ND4: 5′-aggtggttttggctagct-3′, Probe for ND4: 5′-FAM-ccacaccattaattattttaagagcctg-TAMRA-3′. GAPDH was used as an internal control: Forward Primer: 5′-cgtggggctgcccagaacatc-3′, Reverse Primer: 5′-ggatgaccttgcccacagcct-3′, Probes: 5′-FAM-ccctgcatccactggtgctgcc-TAMRA-3′. All primers and probes were obtained from Invitrogen (Shanhai, China). The real-time PCR reactions were performed in triplicate for each sample. qPCR was performed in TaqMan 7900 HT Fast Real-time PCR System with probe and primer concentrations of 250 and 300 nM, respectively, in the final PCR reaction mix. Cycling variables were: 5 min at 95°C and then 50 cycles of 15 s at 95°C and 1 min at 60°C. Controls were performed under the same conditions. Data was analyzed using Microsoft Excel software.

### Statistical Analysis

All results are presented as mean ± SEM. Nonparametric Wilcoxon Test was used to compare the fold change of mtDNA copies. Statistical software PASW statistics 18 was used in this study and a value of *P*<0.05 was considered statistically significant.

## Results

### 1. Short-term Exposure to Nucleoside Analogues did not Affect mtDNA Levels of Mouse Cerebral Neurons

**Figure 1:**
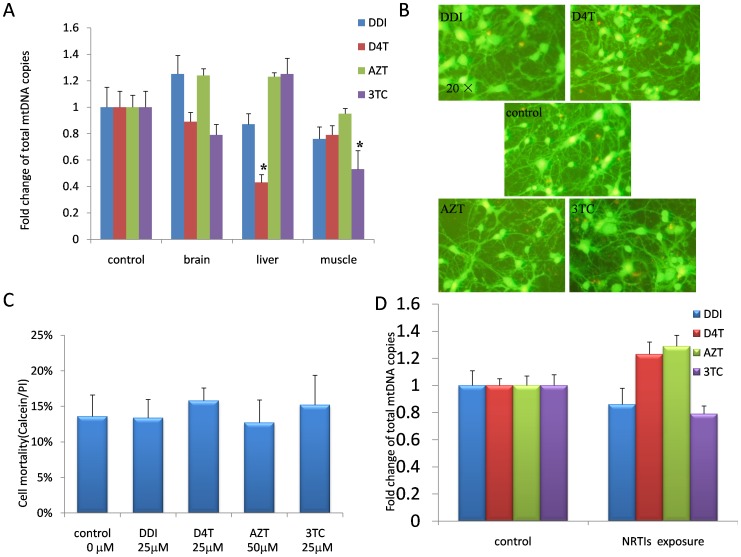
Short-term exposure to nucleoside analogues did not affect mtDNA levels of mouse cerebral neurons. A. In the mice treated with D4T (50 mg/kg), AZT (100 mg/kg), 3TC (50 mg/kg) or DDI (50 mg/kg) for one month, mtDNA loss was not increased in brain, livers (except for D4T ) and muscles (except for 3TC) as measured by COXII specific primers and probes. B. Primary cultural cortical neurons exposed to 25 µM D4T, 50 µM AZT, 25 µM 3TC, or 25 µM DDI for seven days (Calcein/PI uptake test). C. The cell mortality of primary cultural neurons exposed to different NRTIs. D. Increased mtDNA loss was not found in four nucleoside analogues exposed cultural neurons as measured by COXII specific primers and probes (“*” denoted *p*<0.05).

It is well known that long-term exposure to nucleoside analogues can result in the mitochondrial damage in liver and muscle. But the mitochondrial toxicity of short-term exposure to nucleoside analogues was not confirmed. In this study, we firstly tested whether mtDNA loss occurred in the tissues of cortex and liver and muscle from mice treated respectively with different nucleoside analogues for one month. It was showed that although mtDNA loss was found in the D4T exposed mouse livers and the 3TC exposed mouse muscles, the mitochondrial toxicity of other nucleoside analogue did not seem to reach the threshold of mtDNA loss in the livers and the muscles (Fig. 1A). But, in the cerebral cortex, one month nucleoside analogue exposure did not affect mtDNA copies. In order to further investigate the neuro-mitochondrial toxicity of nucleoside analogues, primary cortical neuronal cultures were performed in this study. Neurons are non-proliferating cells and the survival of primary cultural neurons is less than 14 days, thus sustained nucleoside analogue exposure only results in short-term mitochondrial toxicity. In this study, 25 µM D4T, 50 µM AZT, 25 µM 3TC, or 25 µM DDI were confirmed to be the appropriated mtDNA toxicity concentrations without inhibiting neuron growth (Fig. 1B, 1C), and increased mtDNA loss was not found in the neurons treated with each of the four nucleoside analogues (Fig. 1D).

### 2. Long-term Exposure to Nucleoside Analogues did not Affect Total mtDNA Levels of Mouse Cerebral Cortex

**Figure 2:**
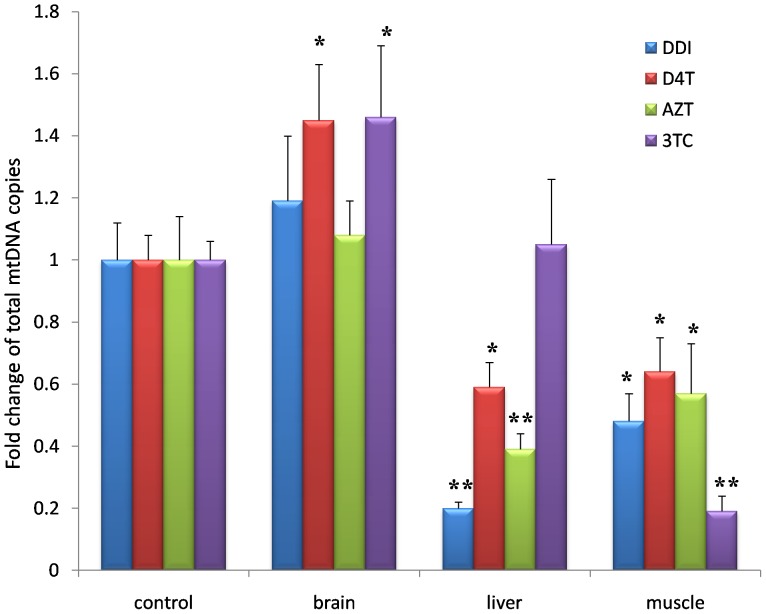
Long-term exposure to nucleoside analogues did not affect total mtDNA levels of mouse cerebral cortex as measured by COXII specific primers and probes. Compared with control, total mtDNA copies significantly decreased in livers of the mice treated with D4T (50 mg/kg), AZT (100 mg/kg) and DDI (50 mg/kg) for four months, but total mtDNA did not change in livers of the mice treated with 3TC (50 mg/kg) for four months. Total mtDNA significantly decreased in muscles of the mice treated with D4T (50 mg/kg), AZT (100 mg/kg), 3TC (50 mg/kg) and DDI (50 mg/kg) for four months. But total mtDNA copies did not lose in brain tisssues of the mice treated with DDI, D4T, AZT and 3TC for four months. Further, mtDNA had even compensatory increased in D4T group and 3TC group (“*” denoted *p*<0.05, “**” denoted *p*<0.01).

Brain, liver and muscle autopsy tissues came from the mice treated with DDI, D4T, AZT or 3TC for four months respectively. Except for 3TC, other three nucleoside analogues decreased the mtDNA levels of liver, while all four nucleoside analogues significantly increased the mtDNA loss of mice muscle (Fig. 2). However, total mtDNA did not decreased in the brain tisssues of mice treated with each of the four nucleoside analogues for four months. Interestingly, total mtDNA even increased in D4T treatment group and 3TC treatment group which probably associated with mitochondrial compensatory response to NRTIs.

### 3. Long-term Exposure to Nucleoside Analogues Increased mtDNA Loss of Mouse Cerebral Cortical Neurons

**Figure 3:**
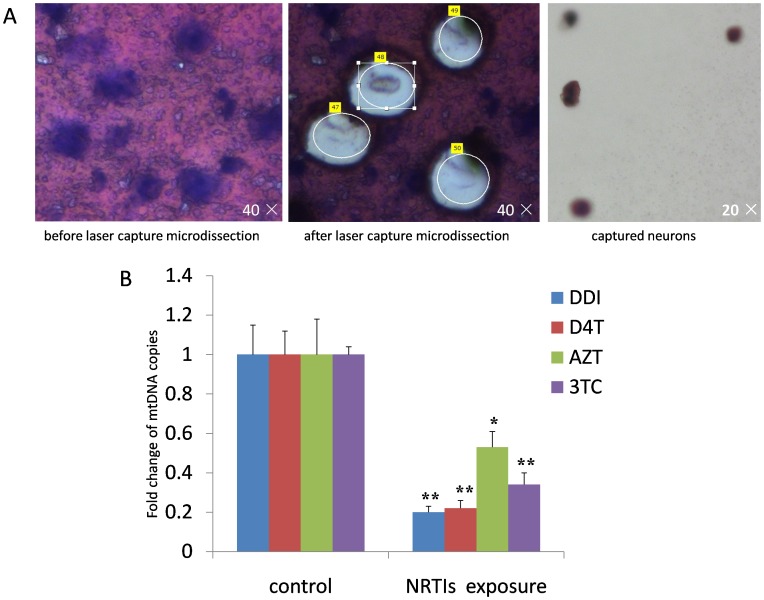
Long-term exposure to nucleoside analogues increased mtDNA loss of mouse cerebral cortical neurons. A. Typical images of Laser Capture Microdissection before and after microdissection (×200 to ×400 magnification). B. Compared with control, mtDNA significantly decreased in captured neurons from brain tisssues of the mice treated with D4T (50 mg/kg), AZT (100 mg/kg), 3TC (50 mg/kg) and DDI (50 mg/kg) for four months as measured by COXII specific primers and probes (“*” denoted *p*<0.05, “**” denoted *p*<0.01).

The brain tissues are made of neurons and glial cells which provide structural and metabolic support for neurons, as well as blood vessels and membranes which are not ordinarily considered part of the brain. There are 100 billion neurons in human brain but slightly more glials to keep the neuron in good condition. Glial cells take up 90% of brain cell space. Therefore, total mtDNA levels of mice cerebral cortex do not give the true copies of neuron mtDNA. Thus, we captured neurons from mouse brain cortex by Laser Capture Microdissection and typical LCM images before and after microdissection are shown in Figure 3A. The data disclosed that mtDNA significantly lost in the captured neurons from brain tissue of mice treated with DDI, D4T, AZT or 3TC for four months (Fig. 3B).

### 4. Long-term Nucleoside Analogues Exposure Mainly Induced Mouse Neuronal mtDNA Major Arc Deletion

**Figure 4:**
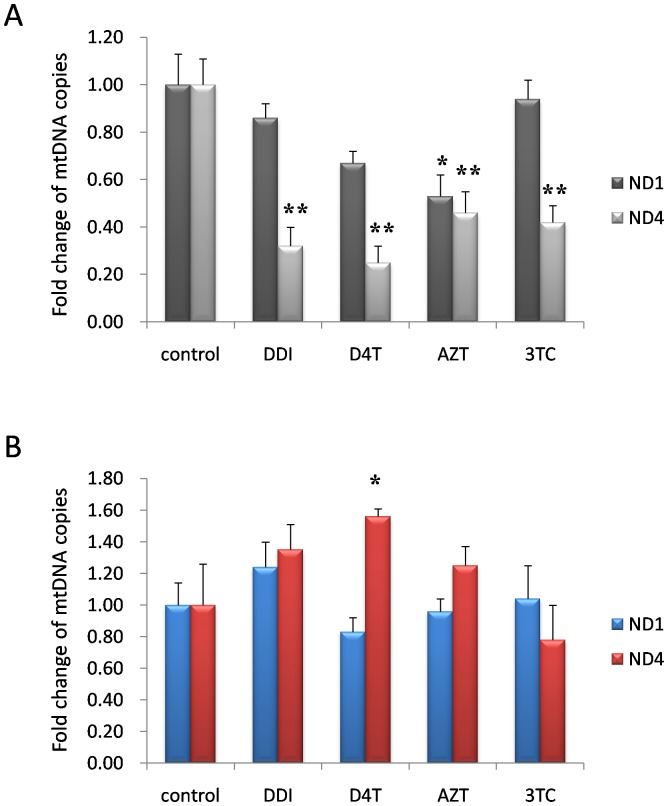
Long-term nucleoside analogue exposures mainly induced mouse neuronal mtDNA major arc deletion. A. The number of captured neuron ND4 (mtDNA major arc) copies significantly decreased in the mice treated with D4T (50 mg/kg), AZT (100 mg/kg), 3TC (50 mg/kg) or DDI (50 mg/kg) for four months. But only AZT group had a decline in ND1 (mtDNA minor arc) copies. B. The numbers of ND4 and ND1 copies from captured glial cells had no significant changes in all four–month-NRTI-treatment groups except for D4T group in which the number of ND4 copies increased instead of reduction (“*” denoted *p*<0.05, “**” denoted *p*<0.01).

The circular mtDNA molecule has a major arc (approximately 11 kb) and a minor arc (approximately 5 kb). Two genes are targeted in the PCR experiments: ND1 from the minor arc and ND4 from the major arc. mtDNA in the major arc has been shown to have twofold more deletions than that in the minor arc in mouse skeletal muscle [13]. In order to determine the mtDNA deletion regions of mouse neurons, we selected ND4 and ND1 to denote the copies of major arc and minor arc (respectively) via realtime qPCR in this study, and nuclear gene GAPDH was used as internal referrence sequence. The results showed that under long-term NRTI exposure including DDI, D4T or 3TC, captured neuron mtDNA deletion mainly occurred in the major arc (ND4) by relative quantitation realtime PCR (Fig. 4A). However, AZT decreased minor arc (ND1) copies as the similar levels as COXII (Fig. 3B.) and ND4. Therefore, AZT induced mouse neuronal mtDNA depletion, but other NRTIs including DDI, D4T and 3TC, mainly induced mouse neuronal mtDNA major arc deletion. Further, the same procedure was performed to detect mtDNA deletion of glial cells. The results showed that the numbers of ND4 and ND1 copies from captured glial cells had no significant changes in all four-month-NRTI-treatment groups except for D4T group in which the number of ND4 copies increased instead of reduction (Fig. 4B). D4T induced high ND4 copies probably contributed to mitochondrial compensatory response to NRTIs.

## Discussion

NRTIs have been associated with a number of dose-limiting toxicities in clinical studies. These include Zidovudine (AZT) induced myopathy, zalcitabine (ddC), didanosine (ddl) and lamuvidine (3TC) induced neuropathy, stavudine (d4T) and fialuridine (FIAU) induced neuropathy, myopathy and lactic acidosis [14]. Transgenic mouse models (TG) in HIV/AIDS research are valuable tools to investigate the pathophysiology of HIV-1 infection and the effects of the antiretrovirals used to treat the infection [15]. In cardiac “dominant negative” murine transgenes models (TGs; Pol gamma Y955C, and TK2 H121N or I212N), NRTIs could increase left ventricular mass [16]. The tissue distribution of phosphorylases responsible for phosphorylation of NRTIs relates to their selective tissue toxicity and tissues highly dependent on oxygen such as the cardiac muscle, skeletal and smooth muscle, the central and peripheral nervous system, the kidney, and the insulin-producing pancreatic beta-cell are especially susceptible to NRTIs associated toxicity [17]. Cellular response to NRTIs exposure showed a complex, time- and dose-dependent pattern over time [18]–[20]. NRTIs contain azido groups that compete with natural thymidine triphosphate as substrates of DNA pol-gamma and terminate mtDNA synthesis, resulting inhibition of nuclear or mtDNA polymerases (or both) and chain termination of replicating DNA at the point of insertion of the nucleoside analogue [14], [21]. In vitro, the triphosphates of NRTIs show the greatest affinity for mtDNA polymerase gamma, a single base DNA repair enzyme [20], [22]. Thus, mtDNA mutation and depletion involve in mtDNA toxicity in HIV-1 infected patients with long-term NRTI therapy [23], [24].

There is evidence that the NRTI-related peripheral neuropathy is due to mitochondrial toxicity [14], [25]. Poorer penetration of antiretroviral drugs through BBB appears to allow continued HIV replication in the CNS [26]. Simultaneously, the cerebral neurotoxicity of antiretroviral drugs was conceived to be compromised due to its poor CNS penetration. Interestingly, NRTIs were found to be able to penetrate other two physical barriers, blood–testis barrier (BTB) and placenta, resulting in mtDNA dysfunction of spermatozoa and infants [27], [28]. One study revealed that 16 weeks of oral treatment with 35 mg/kg per day of ddC could result in myelinopathy in rabbit, exhibiting mitochondrial alterations in Schwann cells of sciatic and tibial nerves and dorsal root ganglia [29]. In another study, mitochondria in fetal patas monkey cerebrum appeared to sustain moderate damage when giving a human equivalent daily dose of AZT during the last half of pregnancy, while its cerebellum mitochondria were not effected [30]. Our results showed that the mitochondrial toxicity of NRTIs in liver and muscle cells was obviously earlier than that of neurons. *In vivo*, Davison reported that no depletion of brain mtDNA was found in eight HIV-positive patients receiving AZT [31]. Schweinsburg disclosed that lower N-acetylaspartate (NAA; sensitive to alterations in mitochondrial integrity) was measured in frontal lobe white matter of HIV+ individuals taking NRTIs using proton magnetic resonance spectroscopy [32]. We previously found mtDNA mutation and loss in the cerebral cortex from autopsy of AIDS patients with HAART treatment even though the correlation of mitochondrial dysfunction and NRTIs needed to be further investigated [12].

Laser capture microdissection (LCM) is a particularly useful tool for recovering small cell samples and even enables researchers to collect individual cells from tissue sections. This method facilitates the separation of histological different cells so that proteins, DNA, or RNA from these cells can be analyzed in isolation from the surrounding unwanted cells [33]. Kohler found decreased mtDNA abundance with tenofovir in transgenic mice model using LCM [34]. In our study, LCM revealed the neurotoxicity of NRTIs. Further, it seemed that the mitochondria of glial cells which took up 90% of brain cell space was not damaged by NRTIs even if additional laboratory are needed to confirm it.
